# Meta-Optics for Optical Engineering of Next-Generation AR/VR Near-Eye Displays

**DOI:** 10.3390/mi16091026

**Published:** 2025-09-07

**Authors:** Junoh Lee, Sun-Je Kim

**Affiliations:** School of Electrical Engineering, Soongsil University, 369, Sangdoro, Dongjak-Gu, Seoul 06978, Republic of Korea; pyock2@soongsil.ac.kr

**Keywords:** metasurface, meta-optics, augmented reality, virtual reality, wearable display, optical engineering, near-eye displays

## Abstract

Meta-optics, enabled by metasurfaces consisting of two-dimensional arrays of meta-atoms, offers ultrathin and multi-functional control over the vectorial wavefront of light at subwavelength scales. The unprecedented optical element technology is a promising candidate to overcome key limitations in augmented reality (AR) and virtual reality (VR) near-eye displays particularly in achieving compact, eyeglass-type form factors with a wide field-of-view, a large eyebox, high resolution, high brightness, and reduced optical aberrations, at the same time. This review highlights key performance bottlenecks of AR/VR displays in the perspective of optical design, with an emphasis on their practical significance for advancing current technologies. We then examine how meta-optical elements are applied to VR and AR systems by introducing and analyzing the major milestone studies. In case of AR systems, particularly, two different categories, free-space and waveguide-based architectures, are introduced. For each category, we summarize studies using metasurfaces as lenses, combiners, or waveguide couplers. While meta-optics enables unprecedented miniaturization and functionality, it also faces several remaining challenges. The authors suggest potential technological directions to address such issues. By surveying recent progress and design strategies, this review provides a comprehensive perspective on the role of meta-optics in advancing the optical engineering of next-generation AR/VR near-eye displays.

## 1. Introduction

The demand for immersive and compact augmented reality (AR) and virtual reality (VR) displays has grown dramatically in recent years [1,2,3,4,5,6]. AR display aims to superimpose computer-generated virtual images on the background scene of the real world and project to the human eye [7]. On the other hand, VR display is targeted to project virtually generated images to the user’s eyes while blocking the image of physical word [8]. The current rapid growth of AR/VR display technologies rely on the advancements of computational hardware, sensor integration, and real-time graphics rendering, etc. As these technologies progress, the form factor and optical performance of near-eye displays (NEDs) for AR/VR have emerged as critical challenges that must be addressed to enable truly wearable and consumer-friendly AR/VR devices [1,2,3,4,5,6].

Meta-optics, a next-generation optical element technology leveraging planar arrays of subwavelength meta-atoms to modulate optical wavefronts in free space, has recently emerged as a promising solution to these challenges [9,10,11,12,13,14,15,16,17,18]. Unlike conventional refractive or diffractive optics, meta-optical elements—particularly metasurfaces, two-dimensional metamaterial defined on a transparent substrate—can arbitrarily control the phase, amplitude, and polarization of light within ultrathin form factors, enabling flat and lightweight optical components with high design flexibility [9,10,11,12,13,14,15,16,17,18]. By virtue of this unique capability, meta-optics is expected to replace conventional optical elements and advance a wide range of integrated optical systems. Thus, the field has risen as a key enabler for next-generation AR/VR display systems, particularly in demonstrating eyeglass-type NEDs [19,20,21,22]. To realize practical and user-friendly AR/VR systems, several stringent optical performance metrics must be satisfied, including angular resolution, field of view (FoV), the size of the eyebox, focal cues, transparency, the brightness of virtual images, form factors, weight, and color dispersion, simultaneously [1,2,3,4,5,6].

From the perspective of optical engineering, however, the primary bottlenecks in achieving such systems lie in the complex trade-off relationships between the aforementioned metrics of image quality and the form factor of a system. It has turned out that conventional optical elements, such as refractive lenses and diffractive gratings, cannot provide innovative solutions to overcome the bottlenecks, while meta-optics offers several key advantages in contrast:

- The ability to shape wavefronts in an arbitrary manner within ultrathin thickness;
- Ease of mass production based on semiconductor fabrication technology;
- Advanced optical functionalities such as control of multiple aberrations, high resolution, reduced crosstalk, polarization control, and color dispersion compensation.

These properties make metasurfaces highly attractive for NED applications such as eyepieces, wavefront correctors, collimators, and waveguide couplers.

Nonetheless, meta-optics also faces significant limitations, including narrow bandwidth, trade-off between material absorption loss and modulation capability, large computing power in design optimization, and challenges in fabrication and mass production for large-aperture elements. Consequently, rather than replacing conventional optics entirely, judicious exploitation of meta-optics and combination with digital signal processing would be essential to resolve the sophisticated technological objectives of AR/VR NEDs.

This review provides a comprehensive overview of methods that apply meta-optics to the engineering of optical architectures in NEDs for AR and VR systems. First, we begin by discussing the basic principles of NEDs and meta-optics briefly. Then, recent progress in meta-optic VR displays is introduced, since the basic optical architectures of VR are much simpler than those of AR. Recent progress in meta-optic VR NEDs is then introduced and only the free-space-type NEDs exploiting metalenses as eyepieces are discussed. The following section analyzes meta-optic AR displays by classifying them into free-space and waveguide-based architectures. Particular emphasis is placed on key demonstrations and emerging device concepts with significant practical value. In the Discussion Section, this review also critically examines their limitations and discusses future directions to overcome current challenges in integrating meta-optics into commercially viable AR/VR platforms. Finally, the review is concluded with a summary of key insights and perspectives on the future of meta-optics for AR/VR NEDs.

## 2. Basic Principles of NEDs and Meta-Optics

### 2.1. NEDs: Basic Principles and the Key Parameters

**Figure 1:**
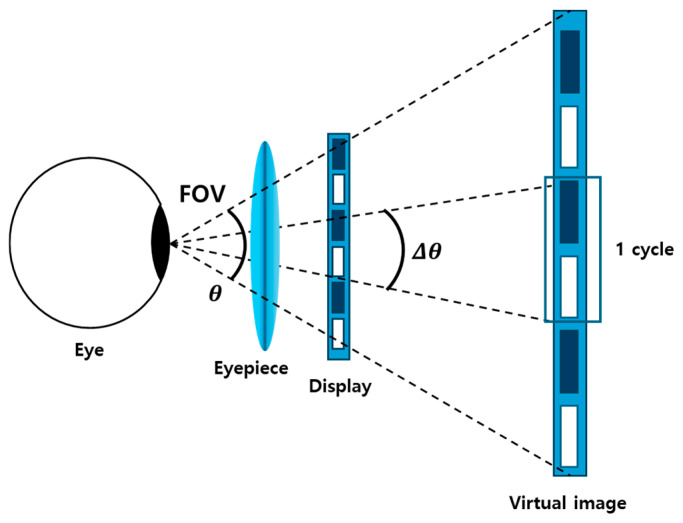
Basic scheme of an NED using an eyepiece lens describing the definitions of angular resolution and FoV.

As can be seen in Figure 1, in the fundamental near-eye display configuration, the eyepiece lens functions as an optical magnifier that projects the image from a display panel into the viewer’s eye, creating a magnified virtual image at a comfortable viewing distance. This simple free-space-type arrangement forms the basis of most AR/VR headsets or glasses, where the primary role of the optics is to enlarge the panel while preserving its visual quality. Within this framework, two of the most critical performance metrics are the angular resolution and angular FoV. The angular resolution quantifies how finely visual details can be distinguished across that field, while angular FoV determines how wide a scene can be perceived by the user. These can be defined by the following equations:

(1) $$Angular\;resolution\;\left(cpd\right)=\;\frac{1}{∆\theta \;\left(deg\right)},$$

(2) $$FoV\left(deg\right)=\frac{N}{2\;Angular\;resolution\;\left(cpd\right)},$$

where *N* is the number of pixels of the display panel and cpd means the cycles per degree.

It can be inferred from Equations (1) and (2) that there exists an inherent trade-off between angular FoV and angular resolution. A larger FoV can be obtained by an eyepiece with larger optical power and it results in enhanced immersion by expanding the visible scene. But for a given panel resolution and pixel pitch, this expansion reduces the angular resolution, making individual pixels more apparent. Conversely, narrowing the FoV concentrates the available pixels into a smaller angle, thus improving perceived sharpness but limiting the extent of the virtual scene. For human visual acuity of 1.0, angular resolution of 30 cpd is required and to satisfy this condition, FoV should be narrowed to about 32Deg using assuming a FHD display panel. This interplay underscores the importance of optical design choices and display specifications when targeting both immersive and visually sharp experiences in near-eye displays.

**Figure 2:**
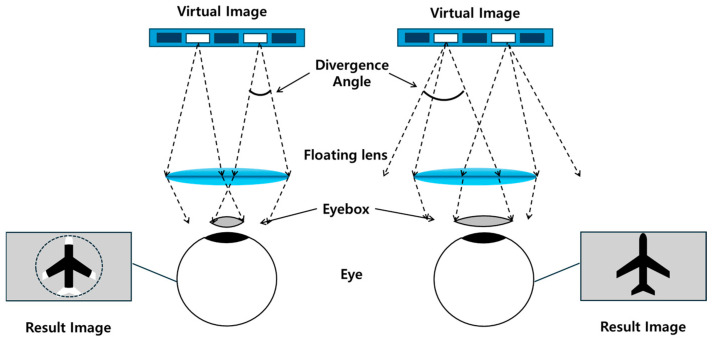
Relation between the eyebox size, divergence angle, and optical power of the floating lens in a basic NED configuration.

The third essential performance parameter in NEDs is the size of the eyebox, which defines the spatial region where the user’s eye can be placed while still seeing the full image. Figure 2 provides a comprehensive graphical description of the definition of an eyebox. In a basic NED scheme, the size of the eyebox is directly influenced by the optical power of the eyepiece lens, as stronger magnification reduces the tolerance for eye position. It is also tied to the pixel pitch of the display panel: finer pixel pitches allow smaller exit pupils and therefore tighter alignment requirements. Achieving a sufficiently large eyebox is crucial for practical usability, ensuring that users can naturally move their eyes and slightly shift the NED without losing image fidelity. Thus, optimizing eyebox size alongside angular FoV and resolution remains a central challenge in the optical design of AR/VR near-eye display systems.

The last key performance metric is the reproduction of focal cues, which play a central role in natural depth perception. Focal cues are governed by the eye’s accommodation response—the adjustment of the crystalline lens to bring objects at different distances into focus [2,3,4]. Conventional stereoscopic displays can provide binocular disparity, but they usually fail to deliver correct focal cues, since the virtual image is projected at a single fixed focal distance. This limitation can compromise both comfort and realism, underscoring the need for optical architectures that more closely match the behavior of the human visual system.

**Figure 3:**
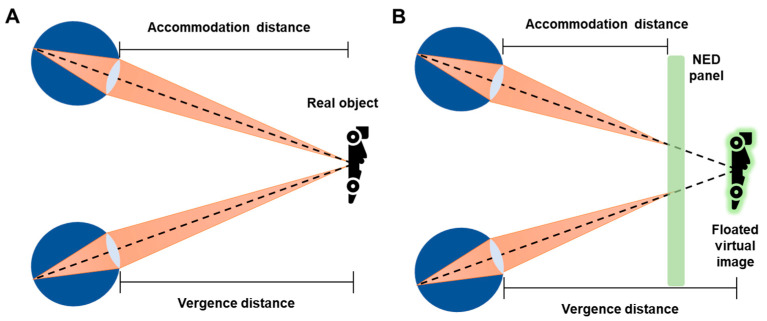
VAC issue of an NED. (**A**) VAC-free stereoscopic viewing case when observing a natural real object. (**B**) VAC-occurring stereoscopic condition when observing an NED panel.

Building on this, one of the most critical perceptual challenges in NEDs is the vergence–accommodation conflict (VAC). In natural viewing, vergence (the rotation of the eyes to align on an object) and accommodation (the lens adjustment to maintain focus) are linked to the same physical distance. In typical NEDs, as described in Figure 3, however, accommodation remains locked at the display panel’s optical distance, while vergence changes according to stereoscopic depth cues. This decoupling produces a mismatch that often leads to visual discomfort, reduced depth accuracy, and fatigue during extended use, making VAC a central barrier to comfortable and realistic 3D presentation.

To address VAC, researchers have explored a range of optical and computational strategies to control the effective focal length of NEDs. Multifocal displays create several focal planes to allow more natural accommodation [15]. Varifocal approaches dynamically shift the focal distance of the virtual image in response to vergence demand [16]. More advanced solutions, such as light field and holographic displays, aim to reconstruct the natural light wavefront, thereby preserving correct depth cues [5]. Recently, foveated rendering combined with adaptive optics has been investigated to balance perceptual fidelity with system complexity [17]. Each of these solutions involves complex trade-offs in hardware design considering the other abovementioned metrics and computational cost, ensuring that VAC mitigation remains a highly important and difficult area of NED research.

### 2.2. Basics of Meta-Optics

As briefly mentioned in the previous chapter, meta-optics is a field aiming to use metasurface platforms to demonstrate ultrathin, advanced optical elements. Optical metasurfaces are ultrathin, planar structures composed of the dense arrays of subwavelength-sized engineered nanostructures—often referred to as “meta-atoms”—that locally modulate the phase, amplitude, and polarization of light [9]. In general, optical metasurfaces are fabricated by patterning high-index dielectric materials such as Si (amorphous, poly-crystalline, and single-crystalline), TiO_2_, SiN, or even glass, by high-resolution lithographic methods such as standard e-beam lithography [9,10,11,12,13,14]. By precisely engineering the nanoscale geometry and arrangement of the meta-atoms, metasurfaces can implement arbitrary wavefront shaping functions in a compact form factor, surpassing the design limitations of traditional refractive or diffractive optics. For example, when designed to focus light, these metasurfaces function as metalenses, capable of achieving high numerical apertures (NAs), aberration correction, and even achromatic performance within an ultrathin profile.

There are three primary mechanisms to build meta-optic elements to encode space-variant phase information into wavefronts in arbitrary manner: the geometric phase, propagation phase, and resonant phase [18]. The geometric phase, often called the Pancharatnam–Berry phase, arises from spatially varying orientations of anisotropic meta-atoms. By rotating identical elements, one can impart a phase shift that depends only on orientation, making this method robust to wavelength variations but polarization-dependent. In contrast, the propagation phase relies on the effective optical path length inside waveguide-like meta-atom structures. Here, the phase is tuned by tailoring the dimensions of each element, offering broadband operation and polarization insensitivity but often requiring relatively thicker or more complex structures. The resonant phase approach, exemplified in Huygens meta-atoms, exploits carefully designed resonances—typically overlapping electric and magnetic dipole responses—to produce strong and abrupt phase shifts. This method allows for high efficiency and compact designs with full 2π phase coverage, but the resonance also introduces narrowband behavior, making performance sensitive to wavelength. In summary, these three mechanisms highlight the distinguished features and design opportunities in design of meta-optic elements: the geometric phase offers simplicity, broad bandwidth, and polarization control ability, the propagation phase supports polarization-independent operation and large degrees of freedom in color dispersion control, and the resonant phase enables efficient but spectrally selective manipulation of phase modulation.

## 3. Meta-Optic VR Displays

**Figure 4:**
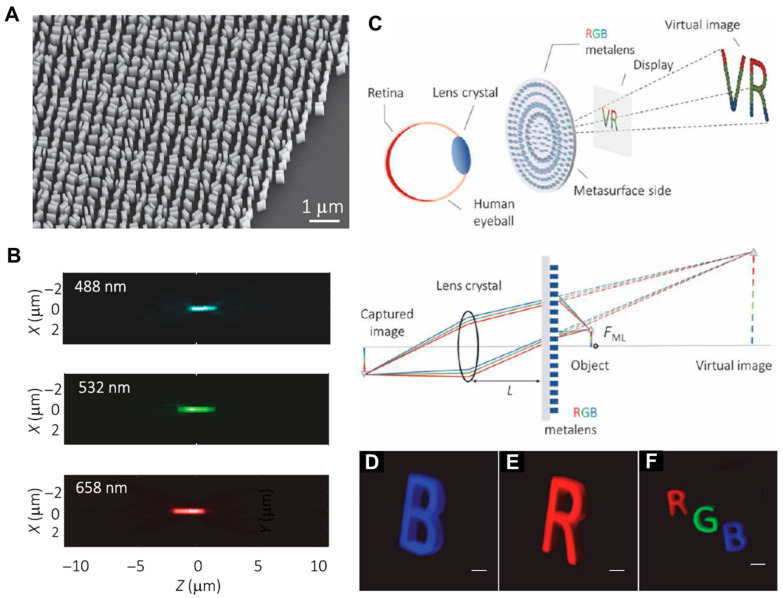
VR display with RGB achromatic metalens based on multi-zone engineering. (**A**) Scanning electron micrograph image of the achromatic metalens. (**B**) Optimized achromatic longitudinal PSFs for the three colors. (**C**) Scheme of the metalens as an eyepiece (magnifier) for meta-optic VR display. (**D**–**F**) Camera-captured images of blue, red, and full-color VR images. Figures (**A**–**F**) are reproduced with permission from Ref. [23], American Association for the Advancement of Science, under a Creative Commons Attribution 4.0 International License.

In this section, several experimental studies of meta-optics-based VR NEDs using eyepiece metalenses are discussed. To provide an immersive display, the eyepiece of a VR NED should provide a wide FoV for RGB colors with thin thickness. Figure 4 presents milestone research suggesting novel meta-optic design solutions for this issue. In 2021, Z. Li et al. from the F. Capasso group at Harvard university reported a novel method to design a singlet achromatic metalens with large aperture and high NA [23]. This work introduces the idea of multi-zone engineering, enabling efficient focusing across the red, green, and blue (RGB) spectrum. Instead of requiring each meta-atom to operate achromatically across the entire visible band, inspired by Fresnel lens design, their design divides the lens into multiple cylindrically symmetric zones, with each zone optimized for constant group delay dispersion and a required group delay profile at the green wavelength. The proposed inverse design method is based on a pre-investigated meta-atom library. This approach circumvents the material and design challenges of full-spectrum achromaticity via a new idea, and they succeeded in fabricating RGB achromatic metalenses with 2 mm aperture with NAs of 0.3 and 0.7 using TiO_2_ meta-atoms (Figure 4A,B).

Using a high-NA mm-scale metalens eyepiece, an ultrathin full-color 3D VR display was developed (Figure 4C–F). Building on their RGB achromatic metalens, the authors demonstrated its application as a meta-eyepiece in a prototype VR display system. The setup employs three laser diodes (RGB) coupled via a fiber scanning technique to generate full-color 3D images. The metalens successfully projects focused, chromatically corrected virtual images to the eye, resolving individual pixels across the RGB spectrum without noticeable chromatic aberration near the optic axis. However, even though the theoretical FoV is nearly 89 deg (~2 sin^−1^ (NA)), a limited FoV of about 10 deg was demonstrated in experiments since inherent severe monochromatic aberrations exist in the singlet metalens and they degrade quality.

**Figure 5:**
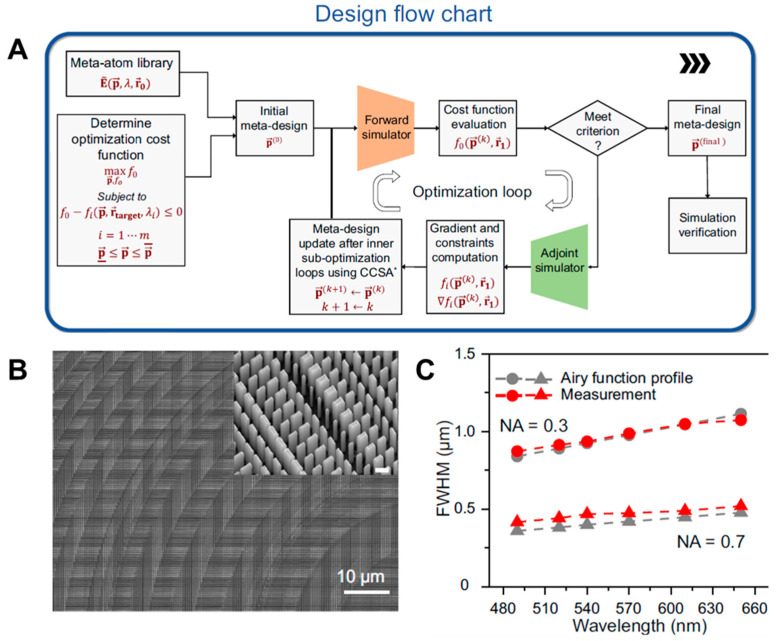
Inverse design of a large-scale achromatic broadband metalens. (**A**) Design work flow. (**B**) An SEM image of the fabricated metalens. (**C**) Broadband achromatic focusing properties obtained from simulation and measurement. Figures (**A**–**C**) are reproduced with permission from Ref. [24], Nature Publishing Group, under a Creative Commons Attribution 4.0 International License.

The next year, the authors from the same group reported improved results for a generalized inverse design framework of large-scale aperiodic meta-optics and its use for VR application. Especially, to achieve broader achromatic bandwidth, larger aperture size, and ease of mass production, while maintaining high NA in the metalens, the algorithm is highly advantageous, and the efficiency and robustness of the algorithm were verified numerically and experimentally (Figure 5A) [24]. The algorithm integrates a fast approximate forward solver—based on Green’s function convolution and a surrogate model trained on RCWA simulations—with an adjoint-based stochastic gradient optimization strategy. This approach supports a high-dimensional design space (up to 10^9^ parameters) with a large-aperture metalens (20 k × 20 k λ^2^) and effectively accounts for fabrication constraints (Figure 5 B) through local field interpolation using Chebyshev regression. The authors evaluated the polarization-insensitive, achromatic RGB metalens with diameters up to 1 cm and NA of 0.3 as well as a mm-scale poly-chromatic metalens (Figure 5C). These lenses achieved high-quality, nearly diffraction-limited focusing with negligible chromatic aberration. It is also worth noting that the fabricated metalens shown in Figure 5B had much simpler meta-atom geometries compared to their previous work so that large scale high-quality fabrication was easier to achieve.

**Figure 6:**
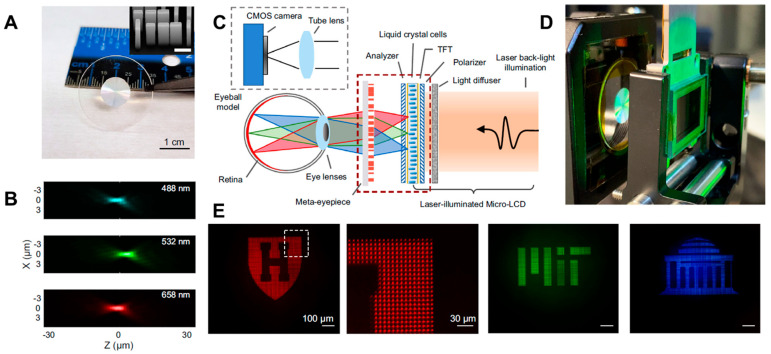
Meta-optic VR display using cm-scale inverse-designed achromatic metalens. (**A**) Photograph and (**B**) achromatic focusing properties of fabricated metalens. (**C**) Scheme and (**D**) photograph of micro-LCD-based VR display setup. (**E**) Measurement results of virtual images. Figures (**A**–**E**) are reproduced with permission from Ref. [24], Nature Publishing Group, under a Creative Commons Attribution 4.0 International License.

To highlight the application potential of their platform, the authors further demonstrated a VR NED integrating their centimeter-scale RGB achromatic metalens as an eyepiece. As shown in Figure 6, paired with a laser-illuminated micro-LCD, this system projects high-resolution RGB images onto the eye with reduced chromatic aberrations and increased focusing efficiency (compared to the previous work [23]). Imaging experiments with static patterns, grayscale scenes, and dynamic video confirmed the system’s capability for full-color VR display with a 60 Hz refresh rate. However, since a singlet metalens eyepiece could not resolve monochromatic (off-axis) aberrations, which is significant to expand the FoV of virtual images, a novel lens design strategy to improve angular resolution or modulation transfer function (MTF) for higher field angles of rays should be studied.

**Figure 7:**
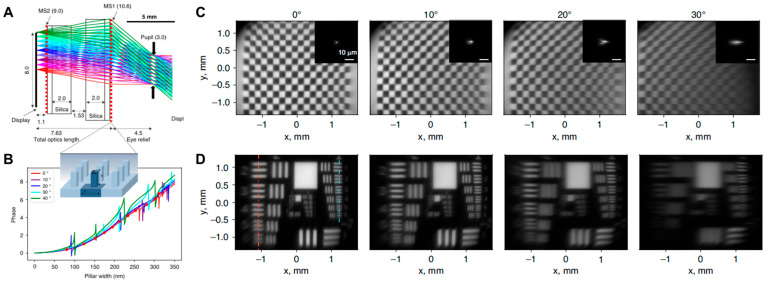
Doublet metlens eyepiece for wide-FOV VR display. (**A**) Ray tracing simulation results of 1 cm-diameter doublet metalens. (**B**) Design of polarization-independent Si_3_ N_4_ meta-atom based on propagation phase, for wide-FOV operation. Experimentally measured images of (**C**) VR checkboard image and (**D**) USAF resolution target. Figures (**A**–**D**) are reproduced with permission from Ref. [25], Nature Publishing Group, under a Creative Commons Attribution 4.0 International License.

Meanwhile, in 2023, W.-Singh et al. from the A. Majumdar group presented a compact VR display architecture based on a doublet metalens system combined with a micro-light-emitting diode (LED) panel designed to achieve wide FoV with high resolution by effectively reducing monochromatic aberrations while maintaining a compact form factor [25]. The system (shown in Figure 7) integrates two cascaded transmissive meta-optics—one convex and one concave—engineered to work in tandem for correcting off-axis aberrations and enabling a wide angular coverage, excepting chromatic aberrations.

Two prototypes were built: a 1 cm-aperture version with an 80° field of view (FoV) and a 2 cm-aperture version with a 60° FoV. The larger 2 cm device was designed under realistic eye conditions (5.4 mm pupil, 15 mm eye relief). Importantly, its total optical track length was 35.7 mm, compared to 43 mm in a commercial refractive triplet, showing that the meta-doublet was thinner and lighter. MTF measurements confirmed that the meta-doublet maintained high image contrast across the full FoV, whereas refractive lenses degraded rapidly at wider angles.

The meta-optics were fabricated using silicon nitride (*n* ≈ 2.0) nanopillars of a 750 nm height and widths ranging from 80 to 310 nm, placed on a quartz substrate (*n* ≈ 1.46). These nanopillars provide a full 2π phase shift with good transmission, optimized using RCWA. The two metasurfaces were mounted with BK7 and fused silica spacers: the first surface acted mainly as a corrective aperture stop, while the second carried most of the focusing power. Design optimization was performed in Zemax OpticStudio at a target wavelength of 633 nm. Fabrication involved electron-beam lithography and plasma etching, with additional discussion of scalable methods like deep-UV lithography and nanoimprint for larger apertures.

When compared to a commercial refractive eyepiece (Edmund Optics RKE, 20 mm aperture, 21 mm EFL), the meta-doublet showed clear advantages. The refractive lens had higher MTF at normal incidence but lost performance beyond a ~20° FoV, limiting its effective use for immersive VR. By contrast, the meta-doublet provided uniform image quality across a 60° FoV, while also being about 17% shorter in total optical length, which directly translates into a more compact and lightweight VR headset design. In short, despite the single color-limited operation without multiple depth information, the proposed metalens doublet platform verifies the idea of solving key limitations of refractive doublets by combining wide FoV, better resolution, and reduced thickness compared to the cascaded refractive optics approach. Table 1 below shows a summary of the three major research articles on meta-optic VR displays discussed so far, in terms of six different performance metrics and the types of display panel. In this table, total system length refers to the length of the VR system from the eye pupil to the display panel (including eye relief).

## 4. Meta-Optic AR Displays

### 4.1. Conventional Architectures of AR Displays

**Figure 8:**
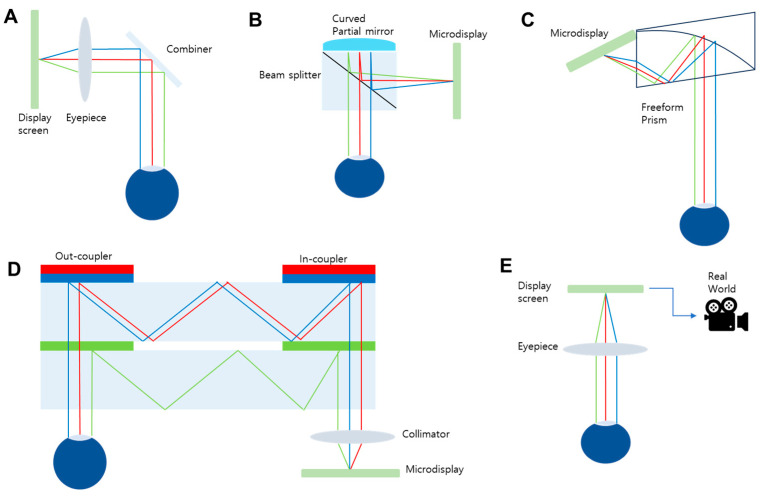
AR display architectures. Schematic diagrams of conventional optical see-through AR display architectures based on (**A**) a combination of a half-mirror and beam splitter, (**B**) a birdbath configuration with a beam splitter and curved partial mirror, (**C**) freeform optics, and (**D**) a waveguide with grating couplers. (**E**) Scheme of video see-through AR display.

Compared to VR displays, designing AR displays poses greater challenges from an optical engineering perspective. This is primarily because AR systems must seamlessly integrate virtual content with the real-world view while maintaining high transparency, minimal distortion, and compact form factors. Unlike VR, where the user’s entire FoV is digitally generated and controlled, AR displays must preserve a clear optical path to the real environment, often through see-through components such as beam splitters or waveguides (Figure 8). This constraint imposes strict requirements on optical alignment, aberration correction, and light efficiency. Furthermore, as in VR, engineering of AR optics must achieve a wide FoV, a large eyebox, and high image quality—all within a thin, lightweight form suitable for wearable use. These compounded demands make AR display design significantly more complex, requiring novel approaches such as freeform optics or meta-optics to overcome the inherent trade-offs.

Figure 8 exhibits various configurations of AR NEDs devised to meet this sophisticated multi-objective design goal. The most basic configuration utilizes a combination of a half-mirror and a beam splitter (Figure 8A), allowing virtual images to be superimposed onto the real-world view with minimal optical complexity. A refined version of this concept is the birdbath configuration, which incorporates a curved partial mirror in conjunction with a beam splitter to improve optical path folding and image focus, albeit at the cost of increased bulkiness (Figure 8B). As can be seen in Figure 8C, freeform optics, another category, utilize specially designed aspheric or non-rotationally symmetric lenses and mirrors to correct aberrations and achieve a more compact design, though they typically require extremely precise alignment and are sensitive to manufacturing tolerances so that mass production cost increases. More advanced architectures leverage waveguide-based systems (Figure 8D), which use grating couplers to inject and extract light within a planar optical substrate. These systems enable lightweight, compact, and highly integrated designs that are particularly suitable for wearable AR NEDs, but they often suffer from limited FoV and large chromatic dispersion. In contrast to optical see-through approaches, video see-through AR systems capture the real world using cameras and digitally combine it with virtual content before displaying it on a screen (Figure 8E). While this method provides greater control over the augmented image and allows for occlusion and lighting effects, it introduces latency and can compromise the sense of direct visual connection to the real environment. Each of these architectures presents distinct trade-offs in optical performance, system complexity, and user experience, highlighting the need for innovative solutions such as meta-optics to overcome current limitations.

From an optical engineering perspective, recent advances in meta-optics offer promising opportunities to address the limitations inherent in conventional AR display architectures, as in VR display systems. In particular, four major roles have emerged in the context of AR displays: (1) metasurfaces as image-combining eyepieces, where they replace bulky beam splitters or curved mirrors to achieve lightweight and compact see-through combiners with tailored FoV and aberration correction [26,27,28,29,30,31,32,33,34]; (2) metasurfaces as relay optics, enabling folded and miniaturized imaging paths that preserve image quality while reducing system volume [35]; (3) metasurface holograms (meta-holograms) as passive display panels [36,37]; and (4) metasurfaces in waveguide-based displays, where they function simultaneously as in-couplers, out-couplers, and expanders—allowing angular and spectral control of light propagation within thin substrates [38,39,40,41,42,43,44,45,46].

In this section, among the four different major roles of meta-optics in AR displays, excepting the third one (meta-holograms), three roles will be introduced with major research results reported recently, by classifying them into two main categories: free-space-type and waveguide-type optics. Among the four roles discussed, meta-holograms are excluded from further consideration, as their practical relevance to AR displays is relatively limited. Instead, they are regarded as being better suited for alternative applications. By contrast, the remaining three approaches are viewed as having more immediate and substantial practical value for the advancement of AR display technologies.

### 4.2. AR Displays with Free-Space Meta-Optics

**Figure 9:**
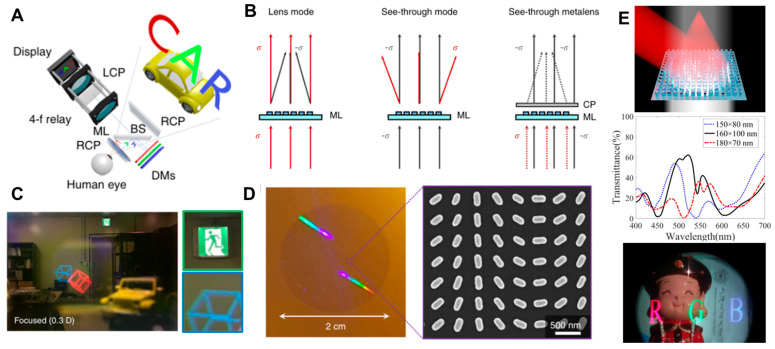
Free-space-type meta-optic AR displays using geometric-phase metalenses as image-combining eyepieces. (**A**–**D**) The first work on a full-color AR display using a multi-functional silicon metalens eyepiece. (**A**) Scheme. (**B**) Concept of multi-functional polarization-dependent metalens. (**C**) Measured AR display scene. (**D**) Fabricated metalens images (photograph and SEM image). (**E**) Full-color AR display based on multifunctional TiO_2_ metalens eyepiece. Figures (**A**–**D**) are reproduced with permission from Ref. [26] and (**E**) Ref. [27], Nature Publishing Group, under a Creative Commons Attribution 4.0 International License.

Figure 9A–D describes a pioneering AR work conducted by G.-Y. Lee et al. with the late Prof. B. Lee of Seoul Natioanl University, in 2018 [26]. This article reports the first demonstration of a free-space AR display system utilizing a metalens eyepiece, marking a significant step toward ultra-compact and lightweight NEDs. The core innovation of this study lies in the design of a see-through metasurface eyepiece lens. The authors engineered a dielectric metasurface composed of anisotropic a-Si nanofins that exploit the geometric phase to achieve both lensing and transparency within the same optical element. By carefully tailoring the geometry and orientation of nanorods, the lens can act as a high-numerical aperture (NA) focusing element for cross-polarized virtual images with circular polarization while simultaneously transmitting co-polarized real-world circularly polarized light with minimal distortion. This dual functionality allows the eyepiece to be placed directly in front of the user’s eye, enabling an ultra-wide field of view (up to 90° in the prototype and theoretically beyond 120° with larger aperture scaling). Fabrication via nanoimprint lithography demonstrated not only the feasibility of large-area, mass-producible metalenses but also high transmission across the visible spectrum, ensuring usability for consumer AR systems.

To realize a functional full-color AR display, the metasurface eyepiece was combined with a projection-based panel system, incorporating beam splitters, polarizers, and dichroic mirrors for color management. A critical challenge—chromatic aberration caused by wavelength-dependent focal shifts—was addressed by using dichroic mirrors to spatially align the red, green, and blue imaging planes, ensuring that all colors were perceived at the same depth. This hybrid strategy enabled simultaneous correction of color fringing while maintaining a wide FoV and high imaging fidelity. Notably, the system achieved immersive AR imaging with both augmented and virtual contents clearly resolved at controlled focal depths, underscoring the practicality of metasurface-based eyepieces for compact, lightweight, and wide-field AR NEDs. Moreover, this approach successfully demonstrated full-color AR imaging with minimized chromatic distortion, thereby addressing one of the major bottlenecks of diffractive optics in wearable displays. However, the system still relied on additional optical elements such as beam splitters for combining real and virtual imagery.

In contrast, in 2022, Y. Li et al. from prof. S.-T. Wu group proposed and experimentally realized a reflective dielectric metalens visor that integrated both an eyepiece and an optical combiner into a single ultrathin device (Figure 9E) [27]. Built on c-Si nanofins arranged to impart geometric phase, this reflective architecture focused oblique incident light for virtual image projection while simultaneously transmitting ambient light for see-through function. Unlike the earlier transmissive system, the reflective visor reduced overall bulk and eliminated the need for a separate combiner, thus offering a more compact and lightweight form factor. The prototype achieved diffraction-limited focusing at 633 nm with competitive efficiency and demonstrated both monochromatic and multi-color AR imaging, validating the practical potential of multi-functional metasurface integration.

The key distinction between the two works lies in their implementation strategies and system integration. G.-Y. Lee et al. achieved wide-field full-color AR with a transmissive metalens but required auxiliary optics for chromatic correction and image combining, highlighting the challenge of dispersion management. Y. Li et al., meanwhile, prioritized system simplification by using a reflective geometry that inherently merges functions, though at the cost of lower efficiencies for shorter wavelengths and limited numerical aperture in the fabricated prototype. Taken together, these works chart complementary paths toward metasurface-enabled AR: one emphasizing dispersion engineering for full-color fidelity and the other advancing multi-functional integration to minimize system complexity and weight.

**Figure 10:**
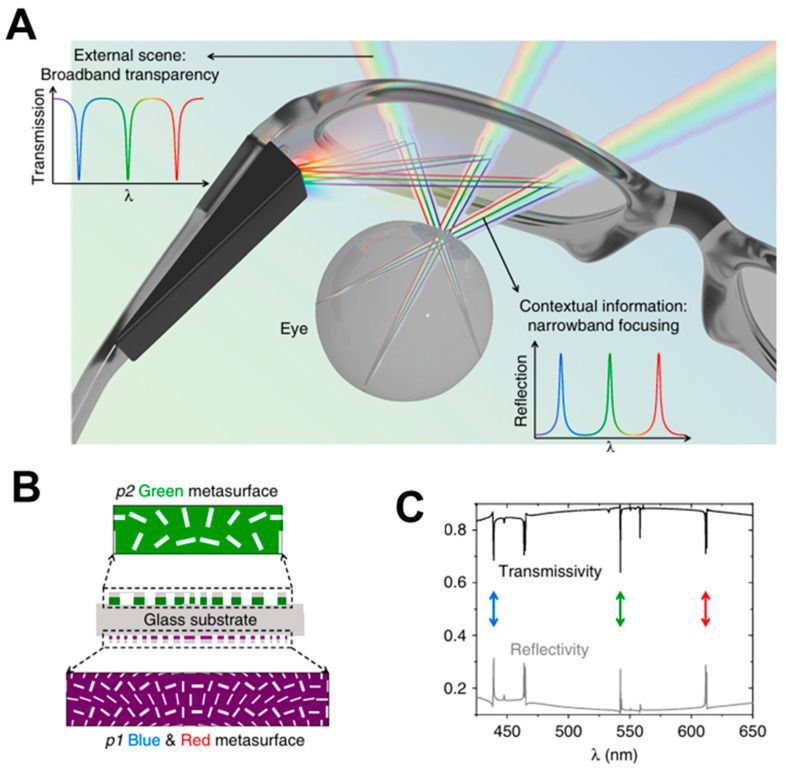
Free-space-type meta-optic AR display based on nonlocal metalens for image-combining eyepiece. (**A**) Schematic diagram of wavelength- and angle-selective full-color nonlocal metalens and its use of image-combining eyepiece in AR glass. (**B**) Scheme of nonlocal metalens doublet designed for full-color operation. (**C**) Optimized simulation results for full-color nonlocal metalens. Figures (**A**–**C**) are reproduced with permission from Ref. [30], Nature Publishing Group, under a Creative Commons Attribution 4.0 International License.

The next work on a multi-functional metalens eyepiece can be seen in Figure 10. In 2022, S. C. Malek et al. from Prof. N. Yu’s group demonstrated a novel eyepiece architecture for AR glasses based on a nonlocal metasurface engineered via quasi-bound states in a continuum (quasi-BIC) (Figure 10A) [30]. Unlike the conventional local-response metasurfaces introduced above, their design exploits collective lattice resonances within periodic TiO_2_ nanofin arrays covered with SiO_2_ anti-reflection layer to achieve nonlocal control over phase and amplitude of transmitted light. The quasi-BIC mechanism ensures a ultra-high quality factor and narrowband resonant filtering, which the authors harness to design a metalens with three spectrally isolated resonance bands corresponding to red (612 nm), green (542 nm), and blue (439 nm) wavelengths. Each band is associated with a distinct quasi-BIC mode engineered by modifying the nanofin geometry and unit cell asymmetry. By spatially multiplexing these RGB-resonant meta-units across the doublet metalens (Figure 10B), they achieve achromatic focusing and beam deflection for full-color AR projection in numerical simulation.

The resulting eyepiece functions as a transparent combiner with high transmission efficiency (>80%) and narrow angular divergence, allowing bright, high-contrast virtual imagery to be overlaid onto the real world (Figure 10C). Compared to geometric-phase metalenses, the nonlocal resonance-based mutli-functional eyepiece design enables precise angular and spectral selectivity while reducing crosstalk. Although the quasi-BIC approach currently relies on narrowband laser illumination and is sensitive to fabrication tolerances, this work paves a novel avenue of a meta-optic eyepiece and represents a significant step toward spectrally multiplexed, full-color, and ultrathin AR meta-optics leveraging collective resonances in metasurfaces.

**Figure 11:**
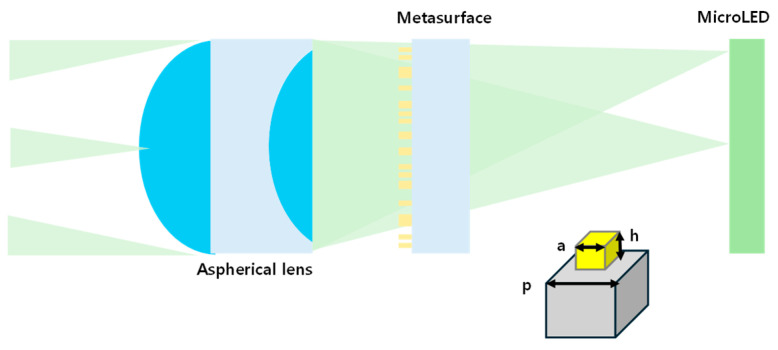
AR display using hybrid refractive (aspheric) meta-optic lens as high-performance relay optics with assistance of AI algorithm for image reinforcement [35].

The second role of meta-optics in AR is acting as a relay optics part. Figure 11 presents a schematic diagram of hybrid meta-optic-based relay optics for an AR display. In 2024, Q. Chen et al. presented a compact AR display system that combines a meta-optics–refractive hybrid relay lens with computational image reinforcement using a neural network [35]. The optical design integrates a transmissive Si_3_N_4_ metasurface—engineered via propagation phase manipulation to impart customized wavefront shaping—with a refractive lens, forming a compact, lightweight relay system capable of correcting monochromatic aberrations and projecting high-quality virtual images. The metasurface is designed to compensate for the off-axis aberrations introduced by the refractive component, and the combined system enables larger MTF over a moderate FoV and improved image uniformity compared to metalenses or refractive optics alone. The glass waveguide with diffractive grating couplers is chosen as an image combiner but more specific description is absent in the paper.

To further enhance image quality, the authors introduce an enhanced super-resolution generative adversarial network trained to restore virtual images degraded by residual off-axis aberrations, without increasing hardware complexity.

The proposed hybrid system achieves high-resolution AR image projection with a moderate FoV (30°), compact system length (7.7 mm; more compact than other studies using metalens eyepieces), high MTF curves (0.5@25 lp/mm for the largest field angle), and minimal optical distortion (<2%). However, a major limitation is that the system is demonstrated using only green color illumination (525 nm LED), leaving its applicability to full-color AR displays unproven. Additionally, while the neural network improves image fidelity, it adds a dependency on computational processing and pre-training, which may limit adaptability in dynamic or unknown environments. Despite these constraints, the work highlights the synergistic potential of physical–optical co-design with computational imaging for next-generation compact AR systems. Table 2 suggested below shows a comprehensive comparison.

### 4.3. Waveguide-Type AR Displays with Meta-Optic Couplers

**Figure 12:**
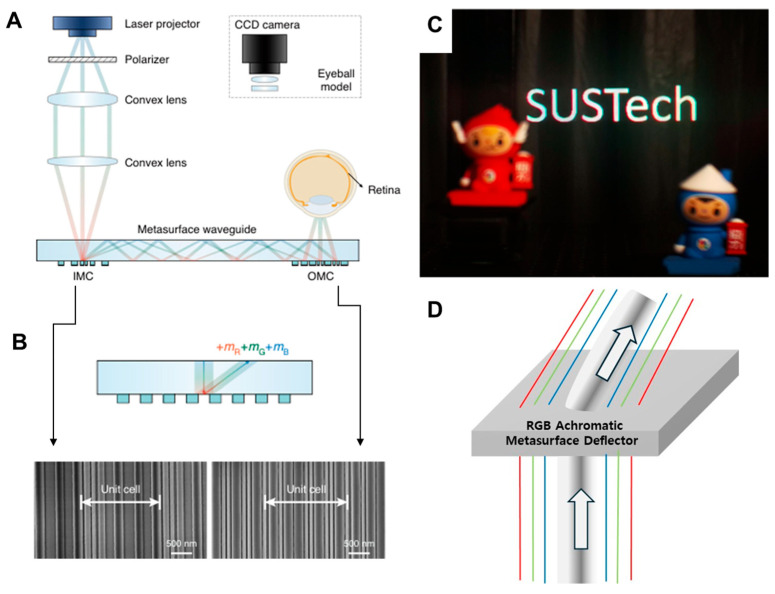
RGB achromatic meta-optic couplers for waveguide AR NEDs. (**A**–**C**) Waveguide-type AR with one-dimensional freeform SiN metasurfaces. (**A**) Scheme of whole system and (**B**) principle of achromatic diffraction with fabricated SEM images. (**C**) Measured AR image. (**D**) Scheme of a-Si:H metasurface deflector with two-dimensional freeform shape [40]. Figures (**A**–**C**) are reproduced with permission from Ref. [39], Nature Publishing Group, under a Creative Commons Attribution 4.0 International License.

With the rapid growth of computational optimization technology in meta-optics, recent advances in freeform metasurfaces have enabled a new class of achromatic optical components, particularly suited for AR applications where compact form factors and color fidelity are essential. The two studies shown in Figure 12 exploit inverse-designed metasurfaces to achieve achromatic beam control across the visible spectrum [39,40]. They share two important features: the use of freeform nanostructures to overcome the limitations of conventional forward-designed unit-cell metasurfaces, and the deliberate choice of diffraction orders for red, green, and blue channels to align deflection angles and thereby suppress chromatic aberrations. These commonalities underline the broader strategy of marrying topology optimization with diffraction engineering to achieve high-performance, fabrication-feasible devices.

The work conducted by Z. Tian et al. represents the culmination of this approach by integrating the two 1D freeform SiN metasurface couplers into a functional full-color waveguide AR system (Figure 12A–C) [39]. The study demonstrates not only achromatic beam deflection with high angular color uniformity but also practical system-level advantages: superior color uniformity, wide field of view exceeding 45°, and a single-layer fabrication process that avoids the alignment challenges of multilayered architectures. Importantly, the authors validate their design in a working AR prototype, showing vivid reproduction of virtual images overlaid on real scenes. This system-level demonstration highlights the practical feasibility of metasurface-enabled waveguides and marks a critical step from device concept to application-ready AR technology.

In contrast, the paper by T. Choi et al. focuses on the development of a multiwavelength achromatic deflector as a fundamental optical building block (Figure 12D) [40]. By employing a single-layer 2D freeform a-Si:H metasurface optimized for distinct diffraction orders of RGB wavelengths, the authors achieve angle-consistent beam steering verified through both simulation and experiments. While the efficiencies achieved—particularly in the blue channel—remain modest, the work provides a rigorous physical framework, including Bloch-mode analysis, to explain and guide the design of achromatic metasurfaces. The study does not extend to a complete AR system but instead establishes the essential device-level principles and fabrication feasibility needed to underpin future coupler integration.

Taken together, these two works illustrate the natural progression of metasurface-based AR research: from the demonstration of an achromatic deflector as a stand-alone component [40] to the realization of a fully integrated achromatic waveguide AR display [39].

A common thread across these three works is the pursuit of achromatic, compact, and efficient coupling strategies for visible-light waveguide displays. All rely on diffraction-order engineering to align RGB beams at identical propagation angles, thereby suppressing chromatic dispersion, and all exploit advanced optimization strategies—ranging from inverse design of freeform nanostructures to STO of periodic metagratings—to overcome the efficiency–uniformity trade-offs that have historically limited metasurface-based couplers. These shared principles underscore the growing consensus that high-performance AR waveguides demand both achromatic functionality and manufacturability within single-layer platforms.

**Figure 13:**
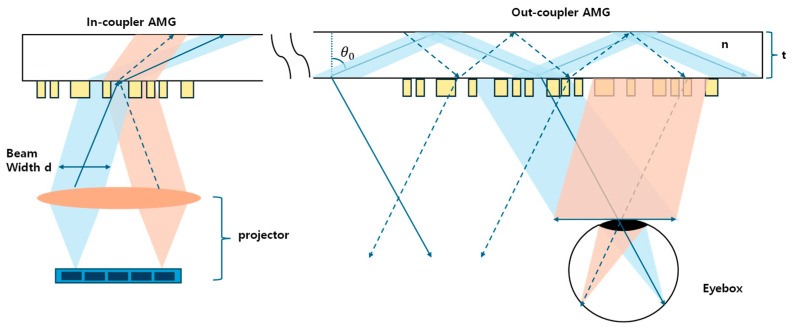
Scheme of the achromatic waveguide display using the 1D freeform meta-coupler. For all fields within the FOV to propagate through the waveguide, the rightmost field, indicated by the black dotted line, must be diffracted into the substrate by the in-coupler achromatic metagrating (AMG) to satisfy the TIR condition [42].

The recent work by S. Moon et al. from Samsung Electronics and POSTECH approaches the same challenge from a complementary perspective, employing achromatic metagratings (AMGs) with 1D freeform shapes rather than 2D freeform surfaces [42]. Based on a SiN metasurface structure nearly the same as that in the work by Z. Tian et al. [39], periodic arrays of metasurface couplers were optimized with an in-house stochastic algorithm using an adjoint method; the authors realized an ultrathin single-layer AR waveguide based on the input and output couplers (Figure 13). The prototype achieves high MTF quality with moderate FoV, a sufficient eyebox, and significantly reduced weight compared to multilayer designs. Importantly, this work emphasizes manufacturability and scalability: the use of metagratings enables simpler optimization, easier subdivision of out-couplers for brightness uniformity, and potential compatibility with nanoimprint techniques for mass production.

**Figure 14:**
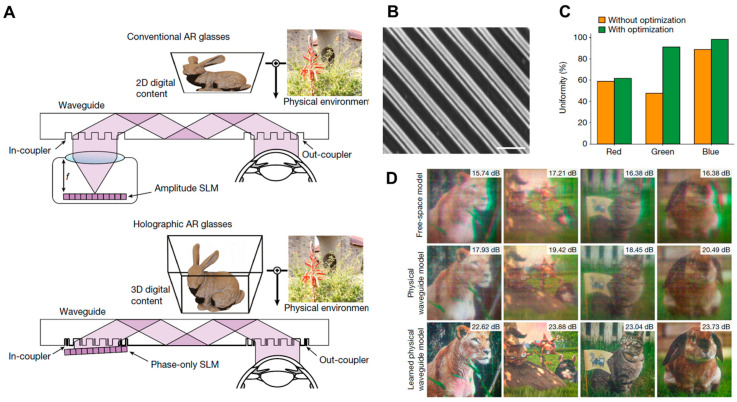
High-performance full-color holographic waveguide AR display based on inverse-designed monolithic SiO_2_ metagrating and physics-informed neural network for full-color holographic display. (**A**) Scheme of proposed holographic AR glass in comparison to conventional one. (**B**) SEM image of fabricated glass metagrating. (**C**) Increased color uniformity by use of inverse-designed metagrating. (**D**) Measurement results. Figures (**A**–**D**) are reproduced with permission from Ref. [43], Nature Publishing Group, under a Creative Commons Attribution 4.0 International License.

The work, described in Figure 14, by Gopakumar et al. from Prof. G. Wetzstein’s group presents a fundamentally different direction compared to the AMG-based study discussed right above [43]. While both employ inverse-designed metasurface couplers for broadband operation, the work by M. Gopakumar et al. tightly integrates the optical hardware with artificial intelligence–driven holographic algorithms. Its couplers, fabricated in high-index SCHOTT glass (refractive index of 1.8), are optimized not only for high diffraction efficiency and angular uniformity but also for compatibility with coherent holographic propagation (Figure 14A–C). This co-design enables full-color 3D holographic content with accurate depth cues, addressing the long-standing VAC in AR near-eye displays (Figure 14D). In contrast to the AMG approach by S. Moon et al., which prioritizes manufacturability, uniformity control, and scalability, the study emphasizes 3D visual quality and realism by fusing nanophotonic design with a computational imaging technique. In particular, from a systems perspective, this work is oriented toward holographic AR, demonstrating compact full-color 3D reconstruction with advanced image formation models calibrated via camera-in-the-loop learning with a physics-informed convolutional neural network model.

Table 3 provides a comprehensive comparison of multiple performance metrics of the three meta-optic AR waveguide displays using metasurface couplers.

## 5. Discussion

With the rapid growth of the meta-optics field, there is increasing optimism that many of the challenges in advanced optical engineering—the primary bottleneck for the commercialization of AR/VR displays—will be progressively resolved. Nevertheless, several critical issues remain to be addressed. Looking ahead, four research directions are considered particularly important for advancing meta-optics in AR/VR applications: (1) establishing scalable approaches for mass production, (2) developing hybrid meta-optics designs that combine the strengths of different optical platforms, (3) innovating integration methods for multiple meta-optic elements within compact display architectures, and (4) enhancing the performance of meta-optic AR/VR systems through computational imaging techniques.

**Figure 15:**
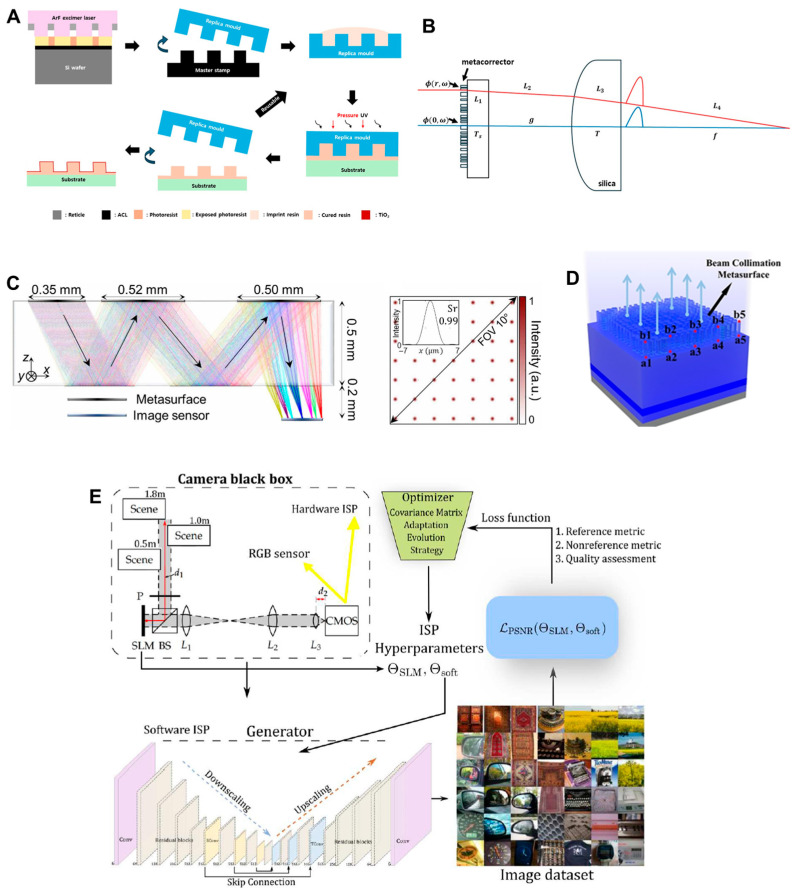
Future research directions of meta-optic AR/VR displays. (**A**) Mass production method of metalens using nanoimprint [48]. (**B**) Design of hybrid refractive-meta-optic imaging system [49]. (**C**) Design of planar optic system with cascaded metalenses. (**D**) Metalens outcoupler for improving light extraction from microLEDs. (**E**) End-to-end co-optimization of software and hybrid meta-optic imaging hardware. Figures reproduced with permission: (**C**) Ref. [53], American Association for the Advancement of Science, under a Creative Commons Attribution 4.0 International License; (**D**) Ref. [55], OPTICA, under a Creative Commons Attribution 4.0 International License; (**E**) Ref. [60], American Association for the Advancement of Science, under a Creative Commons Attribution 4.0 International License.

For scalable and cost-effective fabrication, techniques such as nanoimprint lithography (Figure 15A) and deep ultraviolet photolithography are expected to play a key role in reducing production costs and enabling mass manufacturing of high-performance metasurfaces [47,48]. On the optical design front, hybrid architectures that combine metasurfaces with conventional refractive or holographic optics are gaining attention to overcome intrinsic limitations of metalenses, such as narrow bandwidth and limited efficiency at large apertures (Figure 15B) [49,50,51,52]. Additionally, as can be seen in Figure 15C, the integration of multiple metasurface layers within a single optical module presents a promising path to achieve greater functionality and wavefront control, although it also raises challenges in alignment and fabrication precision [53,54].

Beyond optical components alone, the synergy between metasurfaces and display engine technologies opens new directions for enhancing overall system performance. For instance, metasurfaces can be used to improve the efficiency and radiation patterns of emissions from micro-LED-based displays (Figure 15D) [54,55,56,57], which is critical for achieving high brightness in compact AR form factors. Moreover, combining metasurfaces with SLMs or actively tunable metasurfaces offers the potential for dynamic and reconfigurable optical elements that can enable advanced features such as focal tuning, holographic projection, and adaptive correction [58,59].

The final key research direction lies in leveraging AI-driven computational imaging and pursuing end-to-end optimization of NEDs, wherein both software and hardware are jointly optimized [60,61]. As can be seen in Figure 15E, there have been several reports about introducing creating a flat, lightweight optical system that significantly reduces thickness and volume compared to traditional multi-element lens assemblies, without sacrificing image quality, by combining a planar metasurface with a conventional refractive element. This hybrid design could support broadband, high-resolution imaging with extended depth-of-field, aligning well with the demands of NEDs. Crucially, the work on a miniaturized camera by S. Pinilla et al. adopted a hardware–software co-design approach, wherein the optical front-end and computational back-end are jointly optimized (Figure 15E) [60]. An AI algorithm of convolutional neural network was trained to reconstruct high-fidelity RGB images from blurred and optically distorted sensor data produced by the hybrid lens system. This neural network effectively compensates for inherent aberrations and non-idealities in the physical optics, enabling real-time image restoration. The integrated system exemplifies how computational imaging can offset physical constraints, paving the way for ultra-thin, high-performance AR/VR modules that would be difficult to achieve through optics-only designs.

## 6. Conclusions

In this article, we review AR/VR NEDs and the application of meta-optics in this field, as meta-optics has emerged as a promising technology to address the key performance bottlenecks from the perspective of optical design. To fully unlock their potential for practical deployment, future research must not only advance scalable and cost-effective fabrication methods but also overcome the intrinsic limitations of metalenses through hybrid designs that incorporate refractive or holographic elements. In addition, the development of reliable integration techniques for multilayer metasurfaces, together with system-level optimization in tandem with emerging software algorithms, will be critical to realizing the next generation of meta-optic-based AR/VR NEDs.

## Figures and Tables

**Table 1:** Comparison of performance metrics of meta-optic VR displays.

	Metrics and Type	Total System Length	Aperture Size/NA	FoV of VR	Color	Eyebox	2D/3D	Panel Type
Studies								
Z. Li et al. [23]		N/A (>7 cm)	2 mm/0.3	>10 °	RGB	N/A	3D	Fiber scaning
Z. Li et al. [24]		N/A (>1.6 cm)	1 cm/0.3	N/A	490–650 nm	N/A	3D	Micro-LCD
W.-Singh et al. [25]		35.74 mm	2 cm/0.18	60 °	Red	5.4 mm	2D	Micro-LED

**Table 2:** Comparison of performance metrics of AR displays using free-space meta-optics.

	Metrics and Type	Transparency	Total System Length	Diffraction Efficiency	FoV	Eyebox	Polarization	2D/3D	Panel Type
Studies									
G.-Y. Lee et al. [26] : eyepiece		~ 70% (average)	N/A	29, 6, 5% (RGB)	90 °	10 mm	CP	3D	SLM (Sony)
Y. Li et al. [27] : eyepiece		~30% (average)	N/A	25% (Red)	Small	Small	CP	3D	SLM (Jasper)
S. C. Malek et al. [30] : eyepiece		>80%	N/A	~ 30%	N/A	N/A	CP	N/A	N/A
Q. Chen et al. [35] : relay optics		High	7.7 mm	High (505–560 nm)	30 °	N/A	Independent	2D	Micro-LED

**Table 3:** Comparison of performance metrics of waveguide-type meta-optic AR displays.

	Metrics and Type	Transparency	Diffraction Efficiency	FoV	Color	Eyebox (exit pupil)	2D/3D	Uniformity	WG Thickness
Studies									
Z. Tian et al. [39]		High	8%/2% (In/Out)	45 °	RGB	18.3 mm	2D	>46.3%	1 mm
S. Moon et al. [42]		High (~60%)	20%/1~24% (In/Out)	20 °	RGB	9 mm	2D	>77.5%	0.5 mm
M. Gopakumar et al. [43]		High (>55%)	9, 25, 36% (R, G, B)	11.7 °	RGB	4 mm	3D	>61.7%	5 mm
